# Heart Disease: With Special Reference to Prognosis and Treatment

**Published:** 1900-06

**Authors:** 


**Heart Disease: with Special Reference to Prognosis and Treatment.** By Sir WILLIAM H. BROADBENT, Bart., M.D., F.R.S., and JOHN F. H. BROADBENT, M.A., M.D. Third Edition. Pp. xiii., 420. London: Baillière, Tindall and Cox. 1900.

The third and enlarged edition of this book contains a few illustrations and a coloured frontispiece from a drawing of a case of aortitis.

Sir W. H. Broadbent states that pulmonary and apical systolic murmurs may be audible for days, or even weeks, over the hearts of young men who have indulged in unusual exertion. Apparently writing in ignorance of Dr. Foxwell's theory of the causation of the pulmonary systolic murmur, Sir Wm. Broadbent unwittingly gives it his support. He considers dilatation of the right ventricle is present in these cases, but finds it difficult to explain the presence of the pulmonary systolic murmur.

A chapter is given to fatty degeneration of the heart. The remarks upon the clinical symptoms are interesting, if by fatty degeneration we understand a failure of the heart muscle from various causes occuring after middle life. Fatty degeneration may occur as a result of disease of the coronary arteries, but we have never met with death from such a cause. Fibroid disease of the myocardium is common, and various degrees of dilatation of the heart are associated with chronic Bright's disease or over-indulgence in alcohol, but fatty degeneration of the heart as a cause of death must be very rare, except in acute diseases, such as diphtheria.

The book is a work written by a clinical physician of great experience, and from the clinical standpoint is valuable, containing many interesting observations, such as the one above-mentioned, upon the length of time cardiac murmurs may be audible over the hearts of healthy young adults after exercise. The remarks upon slight degrees of cardiac pain are instructive, but of greater importance are the opinions expressed upon prognosis in the various forms of heart disease. No question in this branch of medicine is more important than that of prognosis, and few, if any, physicians have had the opportunities of Sir Wm. Broadbent for arriving at definite conclusions upon the subject.

